# A novel customized ceramic bracket for esthetic orthodontics: in vitro study

**DOI:** 10.1186/s40510-019-0292-y

**Published:** 2019-10-14

**Authors:** Liu Yang, Guangfu Yin, Xiaoming Liao, Xing Yin, Niansong Ye

**Affiliations:** 1 College of materials science and engineering, Sichuan University, No.24 South Section 1, Yihuan Road, Chengdu, China; 2 State Key Laboratory of Oral Diseases, National Clinical Research Centre for Oral Diseases, West China Hospital of Stomatology, Sichuan University, Chengdu, No. 14, South Renmin Road, Chengdu, China; 3 Department of Orthodontics, Ninth People’s Hospital Affiliated to Shanghai Jiao Tong University, School of Medicine, No. 639 Zhizaoju Road, Shanghai, China

**Keywords:** Customized ceramic brackets, Esthetic orthodontics, Lithium disilicate, 3D printing, Heat-pressing

## Abstract

**Background:**

This study aims to develop a novel process to establish a standardized manufacturing technique of customized esthetic ceramic bracket system (CCB) which could be endowed with individual color and shape to satisfy patients’ individual demands. Material characteristics and mechanical parameters of CCB were evaluated.

**Subjects and methods:**

CCB virtual models were designed individually according to patient’s teeth morphology and clinical demands. 3D printing technology, lost-wax technology, and selected glass-ceramic ingots were employed to fabricate CCB. Scanning electron microscopy (SEM) analyses were performed to characterize the surface morphology of CCB and commercially available brackets (Clarity Advanced; Crystalline VII; Inspire ICE; Damon Q). Static and kinetic frictional resistance (FR), shear bond strength (SBS) and adhesive remnant index (ARI) scores were recorded. One-way analyses of variance (ANOVA) and post-hoc Tukey’s HSD multiple tests were used for statistical analyses.

**Results:**

Multi-color and multi-transparency raw materials facilitated CCB with a wide range of color options and controllable optical properties to satisfy different esthetic demands of individual orthodontic patients. CCB presented same level of FR as commercially available ceramic brackets did. No significant differences (*P* ≥ 0.05) of SBS were observed among CCB-ES (treated silane), Clarity Advanced and Crystalline VII groups, and CCB-E (no silane) attained the highest ARI mean score 3. In the preliminary clinical trial, CCB presented excellent color-matching and shape-matching appearances similar to natural teeth, which made it highly invisible from social intercourse distance.

**Conclusions:**

CCB were demonstrated to be an applicable labial orthodontic bracket system with optimized esthetics and biomechanics. We envision that it would be an ideal alternative for patients who pursue esthetic orthodontic treatment but were not likely to take lingual appliances or clear aligners.

## Introduction

As it happens in contemporary dentistry, orthodontics is undergoing significant development attributed to digital technologies, which influences the diagnosis, planning and the entire process of orthodontic treatment [1, 2]. A novel customized system allows for assessment of changes in 3D and customization of treatment planning brackets, and wires by means of intraoral scanning, cone-beam computed tomography (CBCT), three-dimensional (3D) photography, and computer-aided design and computer-aided manufacturing (CAD/CAM) technologies [3, 4]. At present, a wide variety of customized orthodontic appliances, including metal labial or lingual brackets and clear aligners are developing rapidly and receiving growing attention. Meanwhile, customized appliances have caused tremendous threat to traditional orthodontic brackets [5, 6]. Fixed customized appliances have demonstrated favorable treatment outcome, enhanced patient comfort and significantly reduced chair time and total treatment time [7–10]. Nevertheless, metal labial fixed appliances increase the visual perception on patients’ teeth by the distinct appearance, and there are reports that lingual brackets still induce discomfort in a certain proportion of patients [11, 12].

Ceramic bracket was introduced in 1980s as a more esthetically pleasing alternative to the metallic brackets [13]. Currently available ceramic brackets are almost composed of aluminum oxides and reveal high strength, chemical stability and biocompatibility [14]. With the improvement of ceramic bracket performances, the demand for esthetics orthodontic became the main target [15]. Less visual perception, to which the term esthetic is related, was desired. And the esthetic performances of ceramic brackets are influenced by optical properties of patients’ teeth. To achieve an esthetic performance, brackets should match the tooth color and/or possess proper translucency to allow the underlying tooth color to penetrate through them, making the brackets less noticeable [16, 17]. Technically and economically, it is much harder to fabricate personalized (shape and appearance) ceramic appliances than metallic appliances with conventional forming approaches, whether polycrystalline or monocrystalline ceramic brackets. To our knowledge, no customized ceramic appliances manufacturing technique has been well-established, even though various brands of commercially available ceramic brackets, e.g. Clarity Advanced, Inspire ICE, Crystalline VII, InVu, have been employed for decades.

Additive manufacturing refers to 3D printing technique that enables the manufacturing of a wide range of products with specific properties and shapes that meet patients’ demands [18]. Although 3D printing has achieved some clinical applications in orthodontics [19], surface quality, dimensional accuracy and the mechanical properties still need to be improved in order to allow fabricating customized ceramic orthodontic appliances.

Customization and esthetics are considered as critical factors both in dental restoration and orthodontic filed. All-ceramic materials are well known as ideal dental restoration materials due to the esthetic ability of imitating tooth color. As a representative glass-ceramic material, lithium disilicate material IPS e.max system (Ivoclar Vivadent AG, Schaan, Liechtenstein) reveals a higher fracture toughness and flexural strength [20], and allows dental technicians to customize dental restorations in terms of form and esthetics by heat-pressing or machining technology. Heat-pressing technology allows a more complex shape and less cost for custom fabrication, such as crowns, veneers or bridges, etc. To date, the application of lithium disilicate in the orthodontic filed, however, has not been reported before.

Thus, the aim of this study is to develop a novel process to establish a standardized manufacturing technique of customized esthetic ceramic orthodontic bracket system (CCB), which employed the individual digital design, lithium disilicate materials and heat-pressing technology. The morphology, friction resistance (FR) and shear bond strength (SBS) of CCB were evaluated in comparison with commercially available ceramic bracket products.

## Materials and methods

### The design and manufacture of CCB

**Fig. 1:**
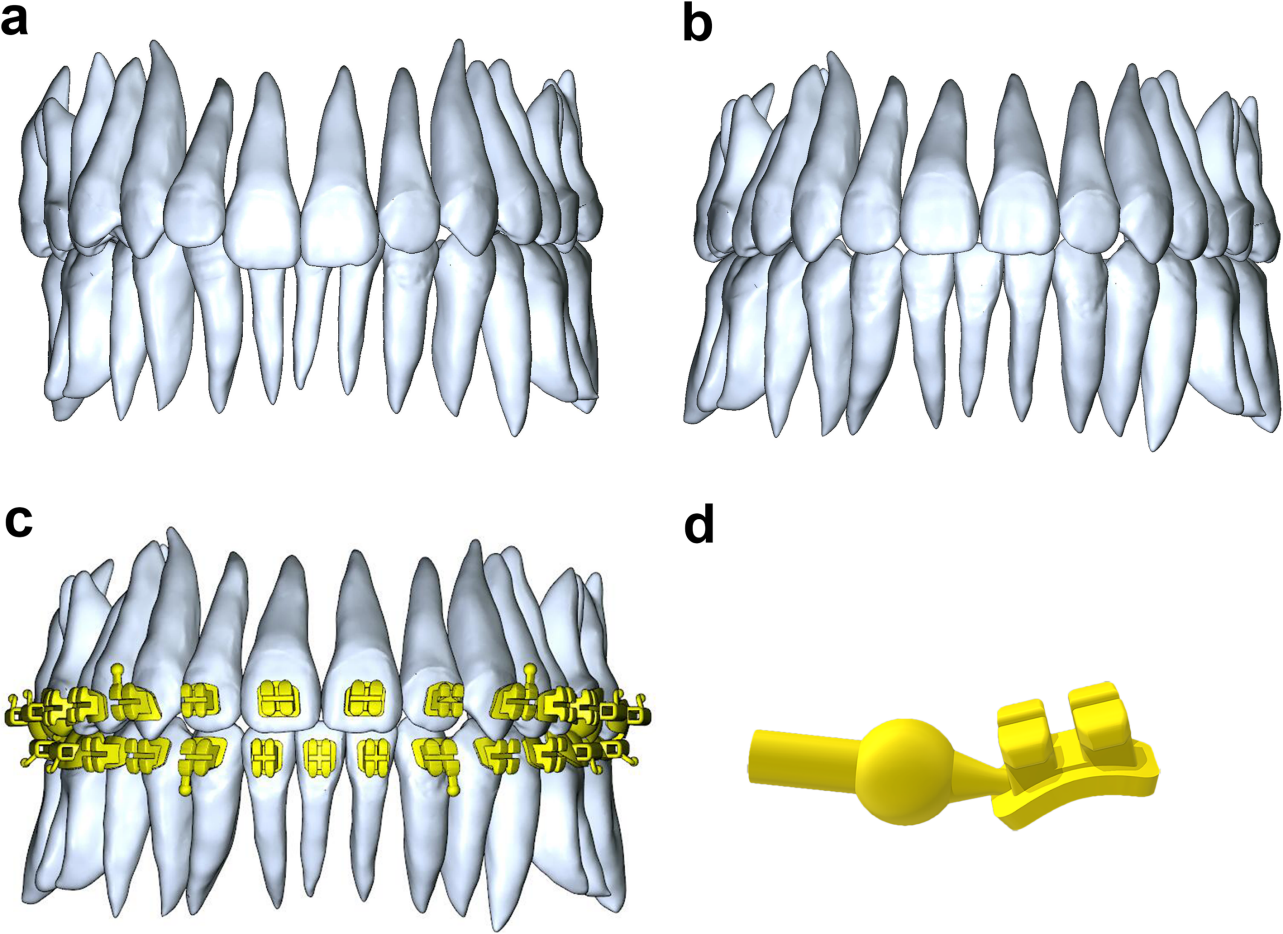
Virtual construction of customized bracket modeling: **a** digital modeling of initial teeth; **b** digital modeling of rearrangement teeth; **c** generating customized bracket bases and body; **d** separating bracket and adding a sprue on side face

Figure 1 shows that the manufacture processes of CCB virtual models involves as follows. Digital models of patient dental arches were obtained via an intraoral scanner (TRIOS 3 Basic, 3 Shape, Denmark) and CBCT (KaVo 3D eXam-i, Kava Sybron, Germany) (Fig. 1a). After virtual teeth arrangement (Fig. 1b), customized brackets which match the morphology of teeth were generated on archwire plane (Fig. 1c). Finally, a sprue was added to each of the bracket model on the side face (Fig. 1d) and the virtual model was exported as a standard triangulation language (STL) file.

**Fig. 2:**
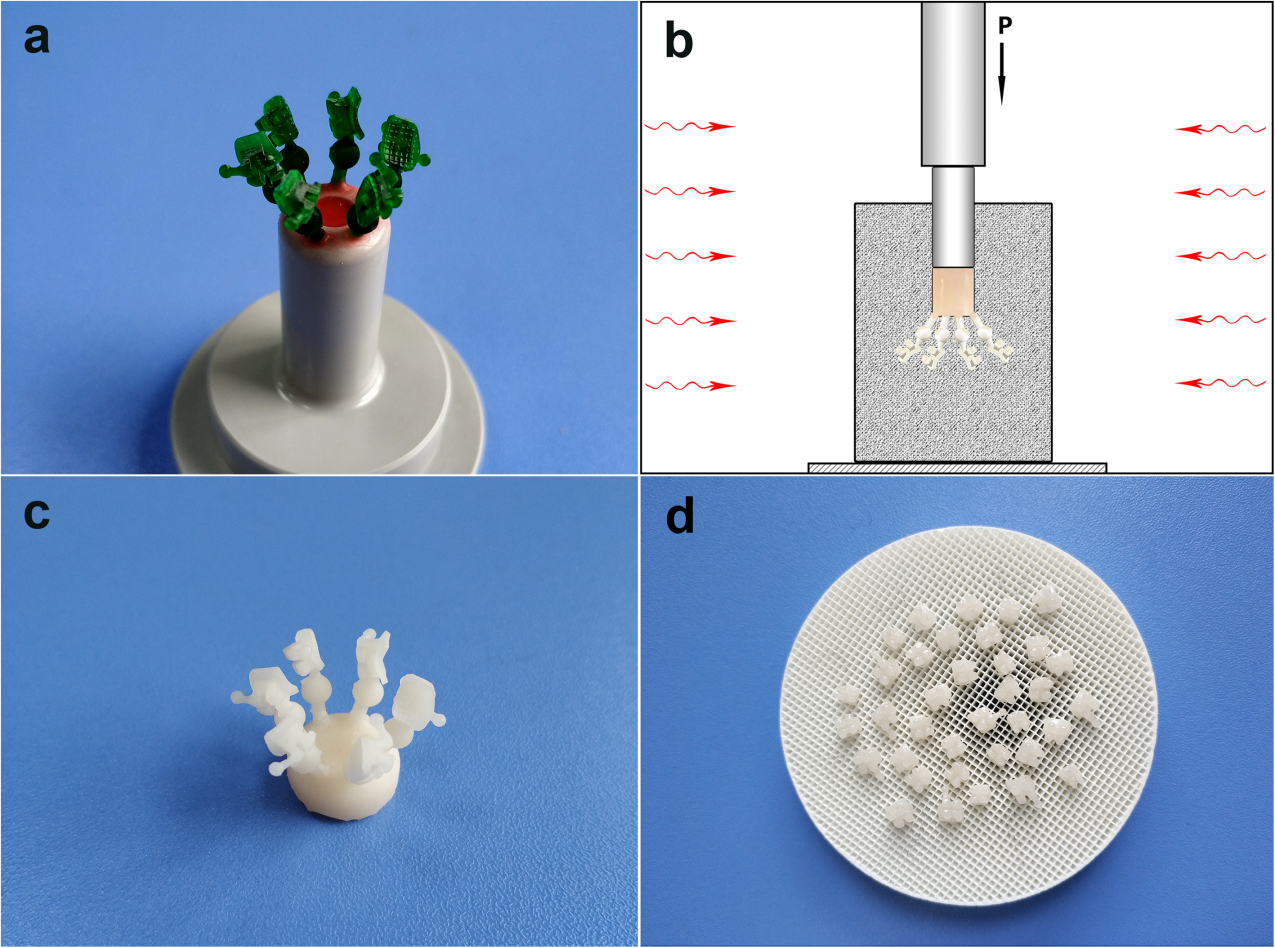
Manufacture processes: **a** wax patterns of customized ceramic bracket; **b** principle diagram of heat-pressing procedure; **c** divesting after heat-pressing; **d** sintering after glazing

High-resolution rapid digital light processing (DLP) technology printer was used to convert the virtual bracket models into wax patterns (Fig. 2a). Glass ceramic ingot (IPS e.max press, Ivoclar Vivadent AG, Liechtenstein), matching the color of patient teeth, was chosen by using a shade guide. Sprueing, investing, preheating, and pressing procedures (Fig. 2b) were carried out according to the manufacturer’s recommendations of dental restorations. Then brackets were divested (Fig. 2c), polished, characterized, glazed, and sintered (Fig. 2d). Finally, ruby oilstone slices with 0.22-in thickness were used to polish the slot of bracket, till a smooth slot looking was achieved.

### Specimen preparation

As shown in Table 1, besides CCB, polycrystalline alumina ceramic brackets Clarity ADVANCED (CA) and Crystalline VII (C7), monocrystalline alumina ceramic bracket Inspire ICE (IC) and a self-ligating metal bracket Damon Q (DQ) were used for in vitro study. All bracket samples were fabricated for maxillary first premolar and contained a 0.022-in slot. Two dimensions of rectangular 0.018 × 0.025-in and round 0.016-in straight stainless steel archwires were chose for FR testing.

**Table 1:** Materials evaluation

Trade name	Materials	Abbr	Slot / wire dimension	Torque	Angulation	Manufacture
–	Lithium disilicate	CCB	0.022-in	− 7°	0°	Self-made
Clarity ADVANCED	Polycrystalline alumina	CA	0.022-in	−7°	0°	3 M/Unitek, CA, USA
Crystalline VII	Polycrystalline alumina	C7	0.022-in	−7°	0°	Tomy Inc., Tokyo, JP
Inspire ICE	Monocrystalline alumina	IC	0.022-in	−7°	0°	Ormco Corporation, CA, USA
Damon Q	Stainless steel	DQ	0.022-in	−11°	2°	Ormco Corporation, CA, USA
Remanium	Stainless steel	16	0.016-in	–	–	DENTAURUM, Ispringen, DEU
Remanium	Stainless steel	18 × 25	0.018 × 0.025-in	–	–	DENTAURUM, Ispringen, DEU

### SEM evaluation

Brackets and archwires morphologies were analyzed by field emission scanning electron microscopy (S-4800, Hitachi, Tokyo, Japan). Low magnification SEM images illustrate the general structure and high magnification SEM images show the microcosmic surface topography.

### Frictional resistance tests

To compare the FR level of CCB with other ceramic brackets, 80 brackets (20 CCB, 20 CA, 20 C7, 20 IC) and 80 SS archwires (40 rectangular 0.018 × 0.025-in archwires and 40 round 0.016-in archwires) were used for this study. Besides, diameter of 0.2 mm stainless steel ligatures and general elastic ligatures were used for archwire ligation. Bracket-wire-ligation combinations were divided into 16 subgroups (*n* = 10) of four bracket types, two wire types and two ligatures. Each bracket was positioned and bonded on a glass slide. The slot was remained in a horizontal position with the long axis of glass slide. A universal testing machine (EZ-LX 1kN, Shimadzu, Kyoto, Japan) was used to investigate the FR in a dry state. Glass slide was fixed to the lower jig of universal testing machine in an upright direction. The angulation between the bracket slot and archwire was 0°, and the archwire was pulled through the slot for 5 mm distance at a crosshead speed of 5 mm/min. For each test of SS ligature (SSL) and elastic ligature (EL), a common bracket was evaluated against the fresh surface of a common wire. The same operator prepared the specimens and performed all tests of this study. Static and kinetic friction forces were measured. The static friction force was determined by the first force peak, while the kinetic friction force was calculated as the mean force from the peak until the end of test.

### Shear bond strength tests

Sixty healthy human maxillary first premolars were prepared and stored at room temperature in 0.1% (wt/vol) thymol solution. Before bonding, roots of teeth were embedded in autopolymerizing acrylic resin with cylindrical mold. Base areas of all brackets were estimated by weighing uniform pieces of lead foil, which was applied to precisely cover the base surface of each bracket. Brackets were treated and divided into six groups (*n* = 10) as following:

Group CCB-E: 5% hydrofluoric (HF) acid gel (IPS Ceramic Etching Gel, Ivoclar Vivadent AG, Schaan, Liechtestein) was applied on the base of CCB for 20 s, rinsed with water spray for 10 s, dried with oil-free air.

Group CCB-ES: Contrasted with group CCB-E, after HF gel etching, all brackets base were subjected to silane coupling agent (Monobond N, Ivoclar Vivadent AG, Schaan, Liechtestein) for 60 s and rinsed with oil-free air.

Group CA, C7, IC, DQ: No treatments were conducted on bracket base except for cleaning up in absolute ethyl alcohol.

The labial surfaces were etched with 37% phosphoric-acid gel (Eco-Etch, Ivoclar Vivadent AG, Schaan, Liechtestein) for 30 s, then rinsed with water spray for 10 s, and dried lightly with oil-free compressed air finally. Adhesive primer (Transbond™ XT, 3 M Unitek, Monrovia, USA) was rubbed onto the enamel surface of all group teeth. Next, brackets were bonded on the teeth surface with a light-cure composite resin (Transbond™ XT, 3 M Unitek, Monrovia, USA), and care was taken to express the composite evenly on all sides. Superfluous adhesive was carefully removed from the periphery of the bracket base with an explorer. All specimens were light-cured with a curing light (Wave length: 420 nm–480 nm) for 20 s. Samples were stored in an isotonic saline solution at 37 °C for 24 h. After 1000 times 5–55 °C thermacycling and 15-s dwell time, shear bond testing was conducted using a universal testing machine (AG-IC 20kN, Shimadzu, Kyoto, Japan) at a crosshead speed of 5 mm/min. After SBS tests, the bond failure bracket bases were examined by SEM. The adhesive remnant index (ARI) score was used to determine the amount of adhesive remaining on the specimen surface [21].

### Statistical analysis

One-way analysis of variance (ANOVA) and post-hoc Tukey’s HSD multiple tests were used to test the significance of differences of FR and SBS values. The results were considered to be significant at *P*-values below 0.05 (*P* < 0.05).

## Results

### Appearance and morphology

**Fig. 3:**
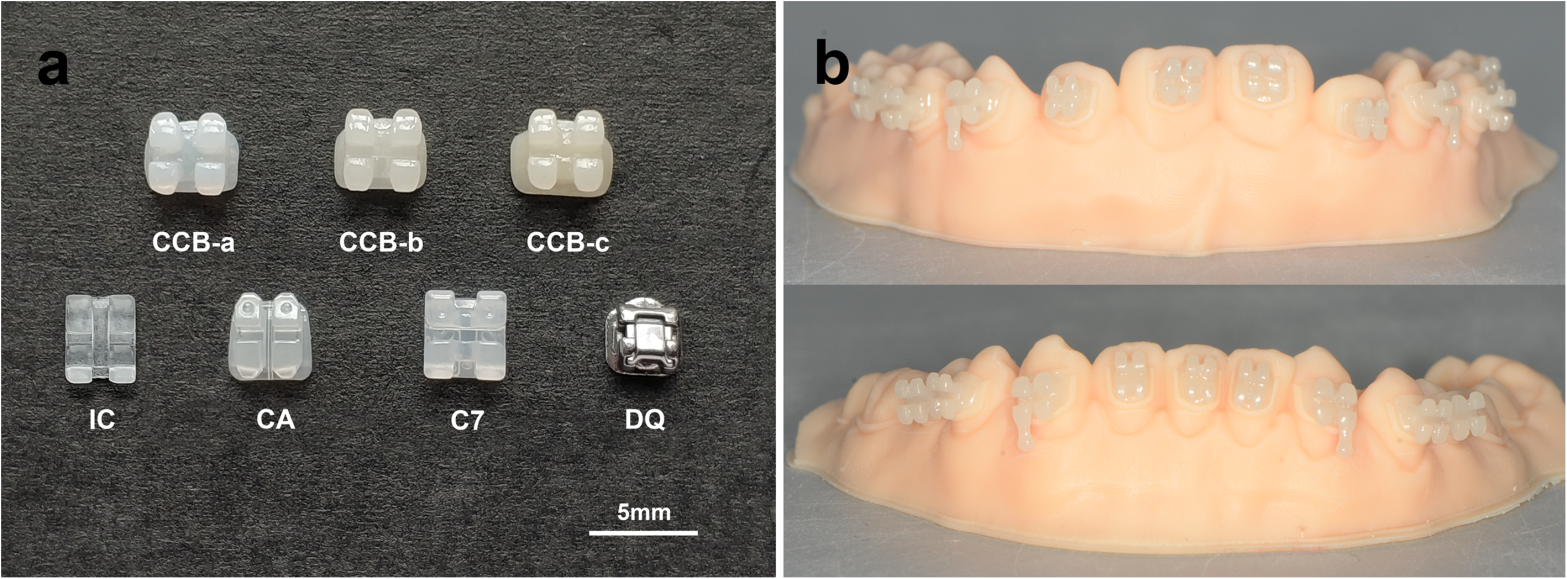
Images of brackets: **a** the different opacity CCB (CCB-a, CCB-b, CCB-c) and commercial brackets (IC, CA, C7 and DQ); **b** customized ceramic brackets bonded on teeth model for fabricating indirect transfer jigs

As shown in Fig. 3a, CCB-a, CCB-b and CCB-c were manufactured with Impulse-O1, HT-A1 and HT-C4 ingots, respectively. With the change of glass-ceramic ingots, the colors of CCB gradually darkened to faint yellow color in natural light, transparency tended to be opaque. The similar appearances were detected among CCB-a, CA and C7. Compared with the other brackets, CCB-b and CCB-c displayed a darker appearance similar to normal natural human teeth color. A set of maxillary and mandibular CCB were bonded on dental cast by experienced technician to ensure accuracy for fabricating indirect bonding transfer jigs (Fig. 3b).

**Fig. 4:**
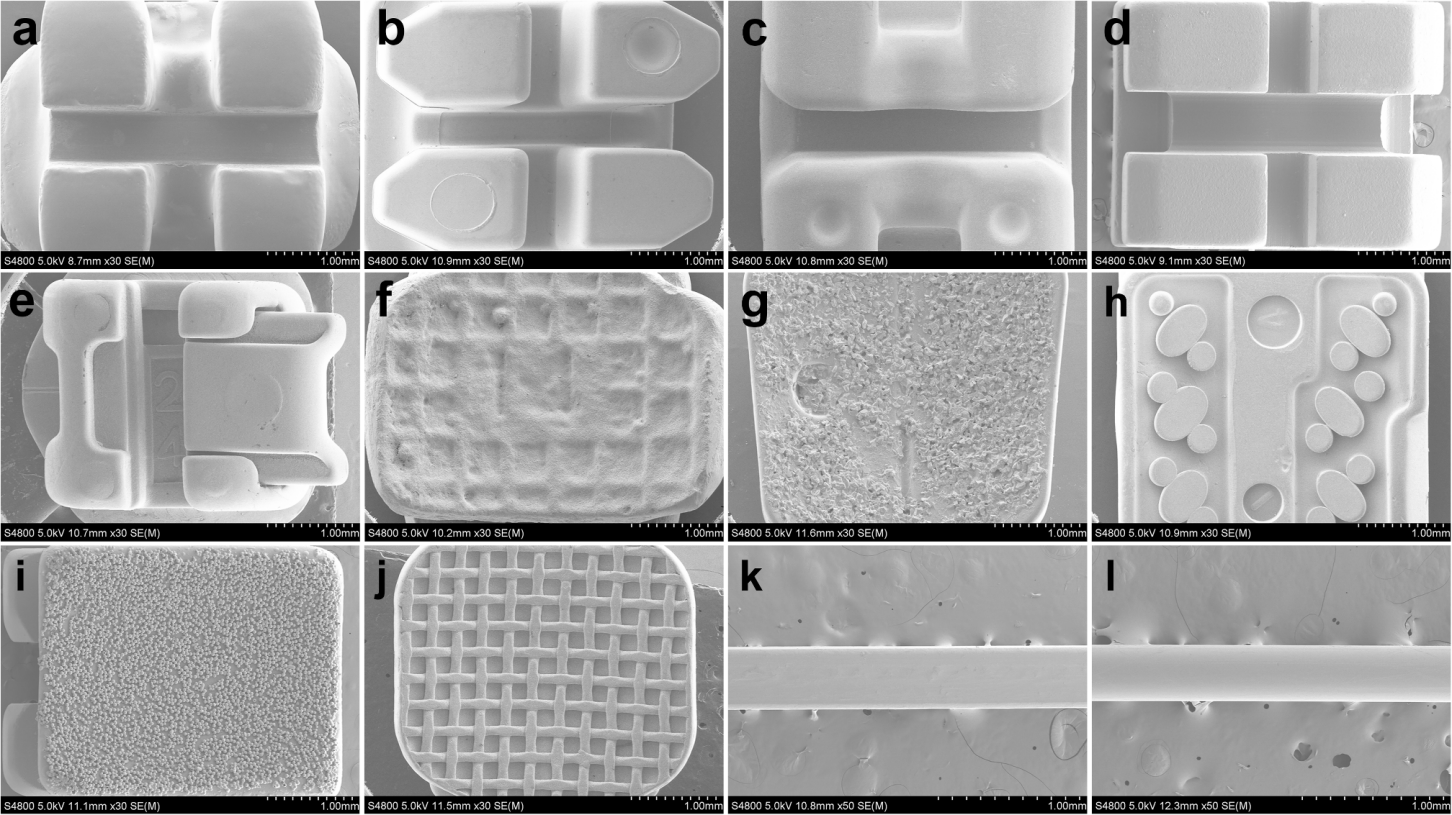
SEM images of bracket bodies, at 30×: **a** CCB; **b** CA; **c** C7; **d** IC. **e** DQ. Bracket bases, at 30×: **f** CCB; **g** CA; **h** C7; **i** IC; **j** DQ. Stainless steel archwires, at 50×: **k** edgewise archwire; **l** round archwire

**Fig. 5:**
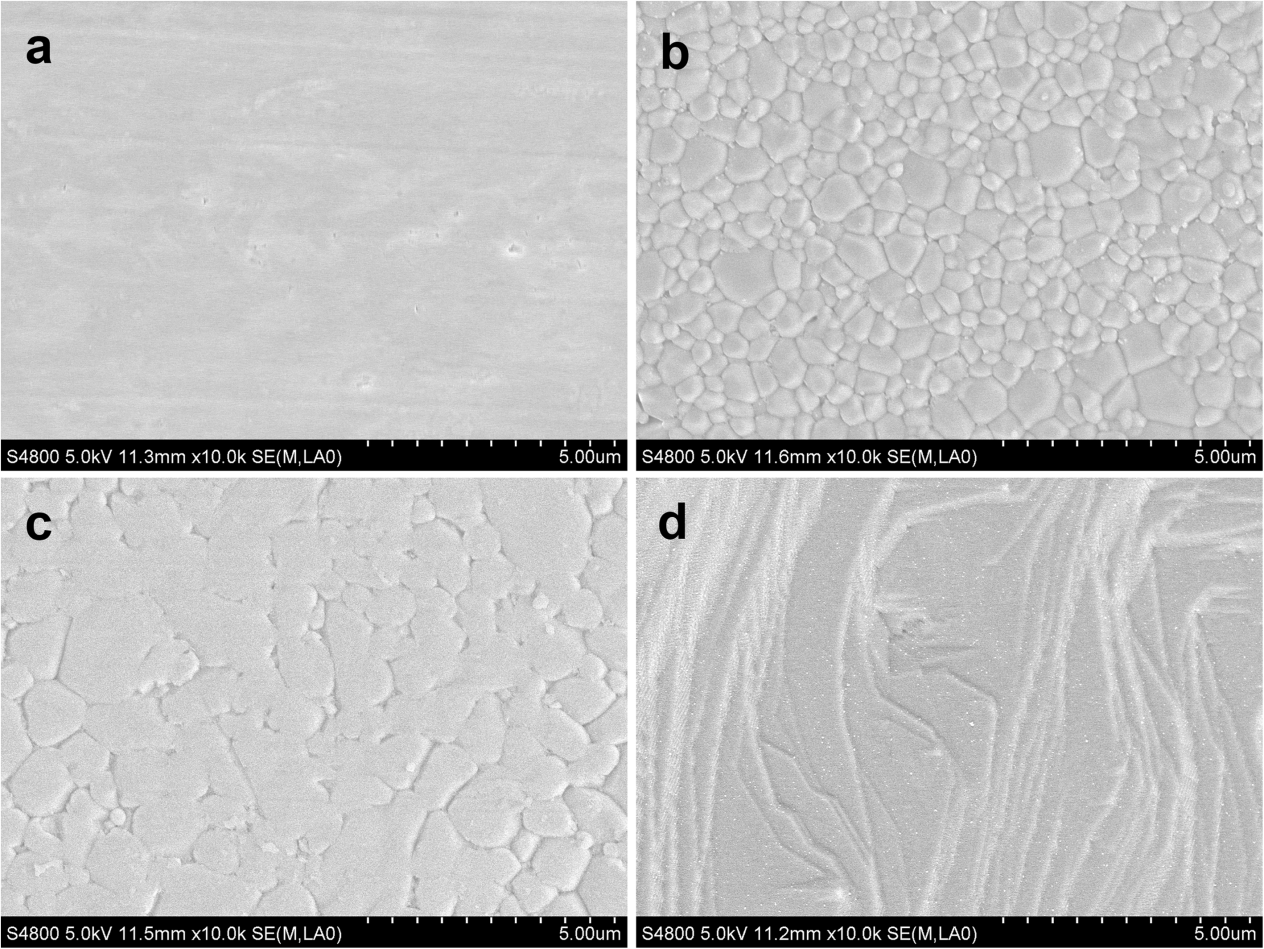
SEM images of ceramic bracket slot, at 10 k×: **a** CCB; **b** CA; **c** C7; **d** IC

Figure 4 shows the different morphologies of brackets and archwires. No obvious defects were found at bracket surfaces (Fig. 4a, e), and various base structures and morphologies were detected (Fig. 4f, j). CCB, CA, C7, IC and DQ base comprised grid pits, randomly oriented sharp crystals, grooves, mass spherical particles and mesh respectively. Smooth surfaces of archwires are observed in Fig. 4k, l. It was obvious that different manufacturing processes and designs resulted in various surface morphologies of slot. After being rasped with ruby oilstone, smooth plane contained some parallel traces and small pits were detected on CCB slot surface (Fig. 5a). CA and C7 slots both revealed smooth and polycrystalline surface, but the grains of CA were smaller and more uniform in comparison with C7 (Fig. 5b, c). Mass constant-thickness step structures were observed on IC slot surface (Fig. 5d).

**Fig. 6:**
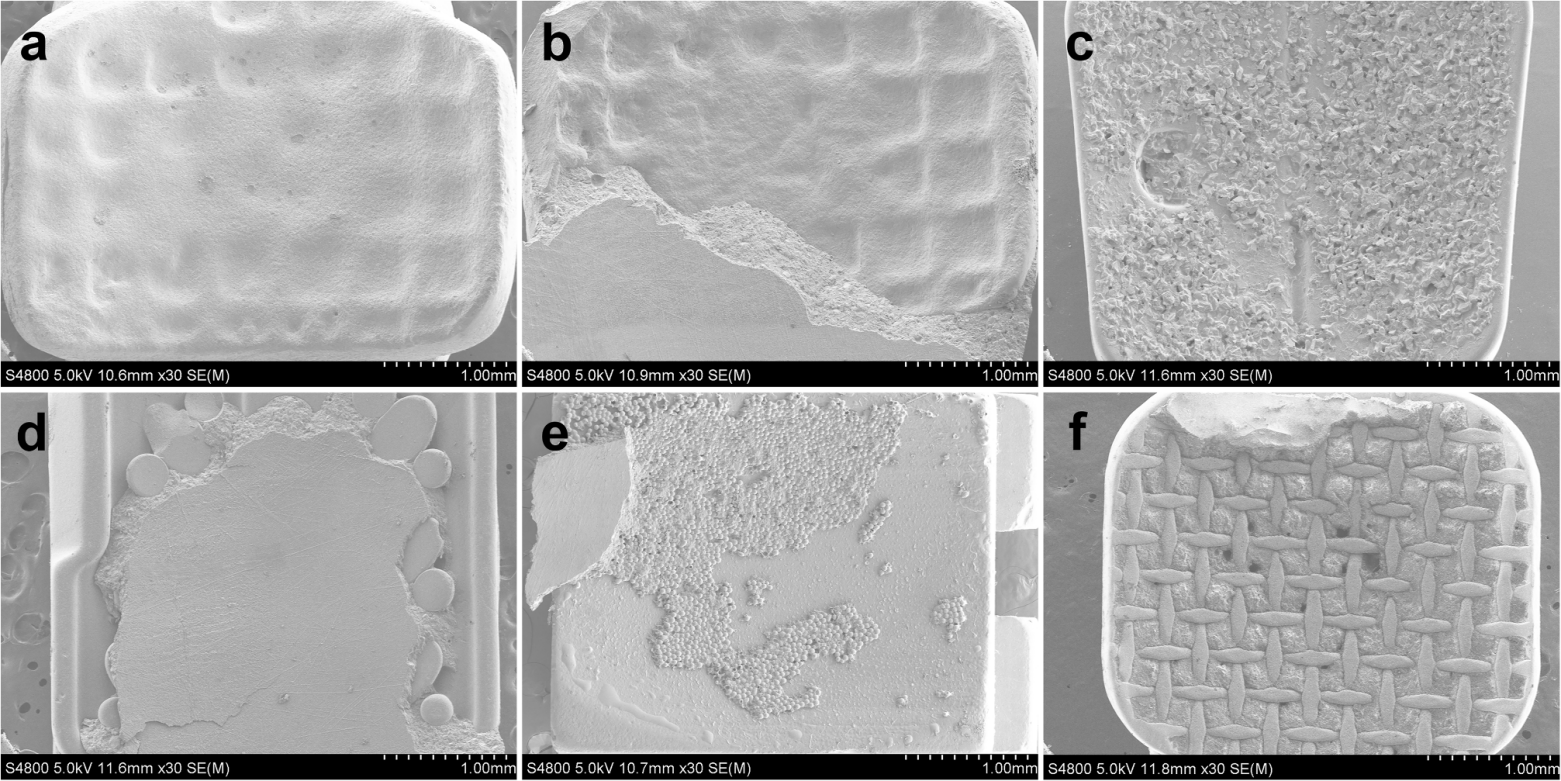
SEM images of debonding bases at 30×: **a** CCB-E; **b** CCB-ES; **c** CA; **d** C7; **e** IC; **f** DQ

**Fig. 7:**
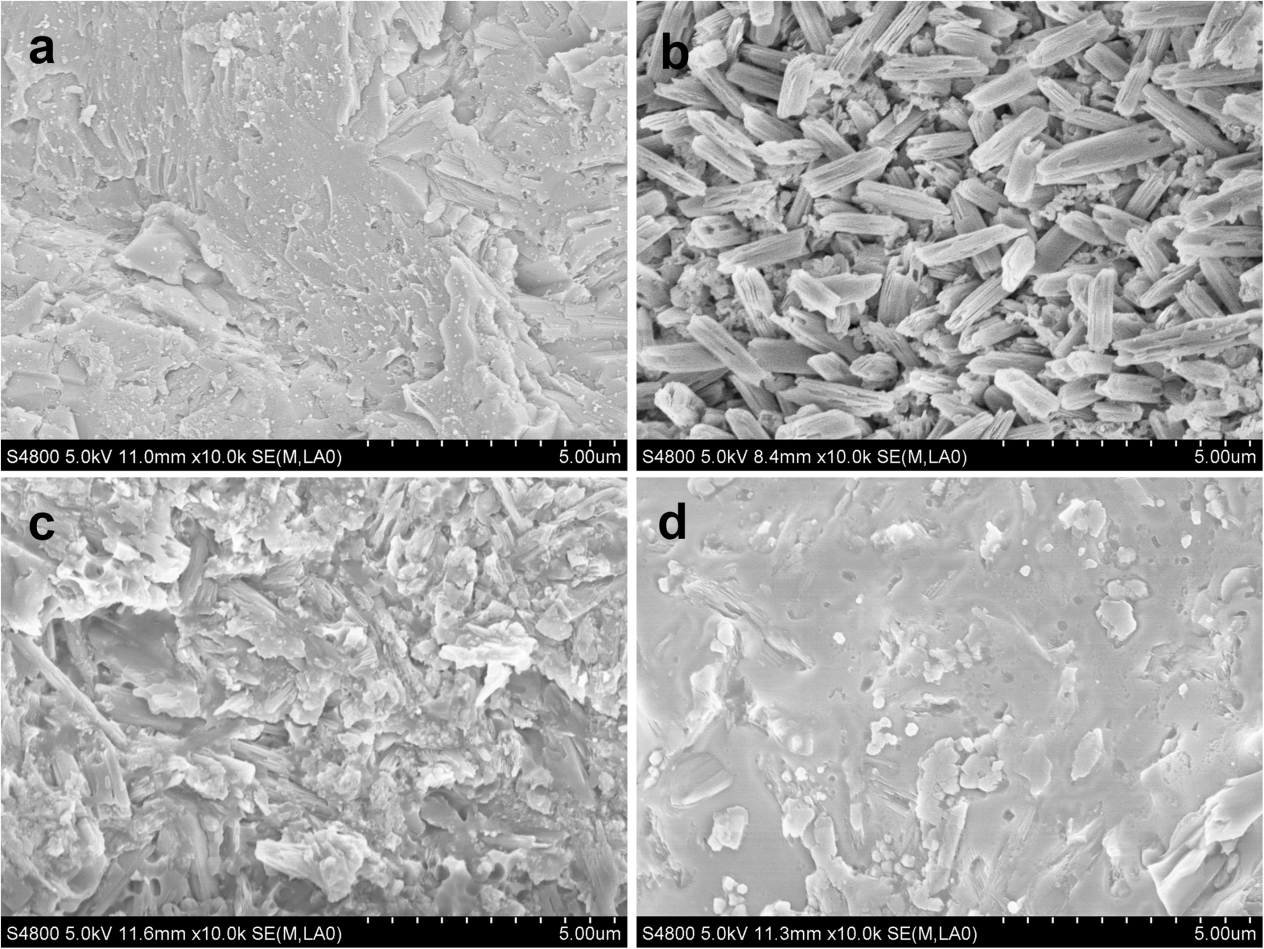
SEM images of customized ceramic bracket base at 10 k×: **a** without etching; **b** hydrofluoric acid etching. And debonding base at 10 k×: **c** CCB-E; **d** CCB-ES

Figure 6 depicts representative morphology of debonding bases. Relatively clear surface was found on CCB-E base (Fig. 6a). In comparison, some large adhesive remnants were detected on CCB-ES (Fig. 6b), Crystalline VII (Fig. 6d), Inspire ICE base (Fig. 6e) and Damon Q base (Fig. 6f). Figure 7a-b display the difference of CCB base before and after HF etching. A rough topographic was detected on initial base (Fig. 7a). After etching, base surface showed a scaffold-like microstructure which comprises some small interlocking needle-like crystals without orientation (Fig. 7b). The break failure of CCB-E occurred at the resin-ceramic interface, and not any effective bonding at resin-ceramic interface was observed but some micro-retentions of adhesives in the irregular pores were detected (Fig. 7c). However, the relatively smooth and clear surface with silane application was found on CCB-ES base, and some crystal fractures and adhesive fragments were also found (Fig. 7d).

### Static and kinetic frictional resistance

A similar static and kinetic FR values are revealed in Table 2. In comparison, the kinetic groups reached lower values. Except group of 16-SSL, no significant differences (*P* ≥ 0.05) were seen between CCB and other commercial brackets. The lowest FR value noticed for IC in all subgroups. For identical bracket-ligature combinations with different archwires, FR data indicated that the lower frictional forces with 0.016-in round archwires than those with 0.018 × 0.025-in rectangular stainless steel archwires. Although C7 showed close FR values between group 16-SSL and 16-EL, the EL of other brackets except CCB presented a higher FR value than SSL with the same dimensional archwire.

**Table 2:** One-way ANOVA comparing different group static and kinetic frictional resistance in Newton

Group	Static frictional resistance			Kinetic frictional resistance		
	Mean	SD	P-value	Mean	SD	P-value
18 × 25-SSL			< 0.001			< 0.001
CCB	0.77^bc^	0.18		0.70^bc^	0.16	
CA	0.72^b^	0.20		0.68^b^	0.18	
C7	0.94^c^	0.15		0.87^c^	0.14	
IC	0.52^a^	0.08		0.50^a^	0.08	
18 × 25-EL			< 0.001			< 0.001
CCB	0.93^b^	0.11		0.90^b^	0.11	
CA	1.00^bc^	0.19		0.89^b^	0.14	
C7	1.14^c^	0.17		1.00^b^	0.17	
IC	0.59^a^	0.05		0.57^a^	0.05	
16-SSL			< 0.001			< 0.001
CCB	0.75^c^	0.17		0.70^c^	0.14	
CA	0.55^b^	0.13		0.52^b^	0.13	
C7	0.53^b^	0.15		0.48^b^	0.14	
IC	0.33^a^	0.03		0.30^a^	0.05	
16-EL			< 0.001			< 0.01
CCB	0.48^a^	0.10		0.44^a^	0.08	
CA	0.65^b^	0.06		0.54^b^	0.07	
C7	0.54^a^	0.05		0.45^ab^	0.05	
IC	0.45^a^	0.08		0.44^a^	0.07	

Mean values with the same superscript letters in common group were not statistically different (P ≥ 0.05)

### Shear bond strength and adhesive remnant index

The mean SBS values and ARI scores are shown in Table 3. Multiple comparison test results indicated that IC group (21.02 MPa) presented the highest SBS value among all groups. There were no statistically significant differences (P ≥ 0.05) in SBS values among groups CCB-ES (15.16 MPa), CA (14.02 MPa) and C7 (16.15 MPa). The Tukey’s HSD test showed that the SBS values of CCB-ES significantly improved (*P* < 0.05) after etching and silane treatments. The highest ARI score 3 was observed for CCB-E group and all brackets keep no residual cement on the base surface. The ARI data indicated that adhesive failures at the ceramic-resin interface were the predominant mode of failure in debonding groups except C7 and DQ.

**Table 3:** Shear bond strength values and adhesive remnant index scores of all tests groups

Group	Shear bond strength			Adhesive remnant index				
	Mean	SD	P-value	ARI 0	ARI 1	ARI 2	ARI 3	Mean
CCB-E	10.21^a^	2.30	< 0.001	–	–	–	10	3
CCB-ES	15.16^b^	2.67		–	1	5	4	2.3
CA	14.02^b^	2.00		–	–	1	9	2.9
C7	16.15^b^	2.56		–	7	2	1	1.4
IC	21.02^c^	2.00		1	–	3	6	2.4
DQ	10.09^a^	1.07		–	4	4	2	1.8

Mean values with the same superscript letters were not statistically different (P ≥ 0.05). ARI 0, no composite remained on the tooth surface; ARI 1, less than 50% of composite remained on the tooth surface; ARI 2, more than 50% of the composite remained on the tooth; ARI 3, all of the composite remained on the tooth, with distinct impression of the bracket mesh

## Discussion

This customized system addressed two problems traditionally associated with labial fixed appliances: first, current metallic customized appliances fail to achieve the esthetic appearance as ceramic brackets do. Second, customized design and manufacture of ceramic brackets are technically limited which adversely affect the development of ceramic brackets. Aiming at these problems, CCB was designed and fabricated by employing CAD technology, 3D printing technology, heat-pressing technology and multi-color lithium disilicate materials.

Virtual treatment planning provides an effective visualization of the final leveling and alignment of the individual arches, so that orthodontists and patient could visually get to know the treatment process and results. Customized design and fabrication not only allows us to reduce bracket thickness to a minimum, but also matches the teeth surface morphology and appearance (color and transparency) to satisfy the specific clinical needs. Besides, the multi-color and multi-transparency system provides a larger tolerance to CCB to imitate various teeth appearances. It is true that customized system does require more time for bracket design and manufacture, but the form-fit properties between the custom bracket base and tooth could achieve a positive lock that makes a correct positioning [22]. Hence, The CCB system is deemed as a promising strategy to provide higher quality of orthodontic treatment by reducing discomfort, chair time and treatment duration and achieving better treatment results.

The high fracture toughness and flexural strength of lithium disilicate materials facilitate the manufacturing ceramic orthodontic brackets. And benefited from lost-wax technique, there are no needs to prefabricate a mold as usual and the duration of fabrication can be reduced significantly. SEM results confirmed that CCB performed a smooth and high integrity surface and slot after the procedures of grinding, polishing and glazing.

In orthodontic sliding mechanics, there are many variables to affect the magnitude of the frictional force [23, 24]. Both static and kinetic friction results indicate that ligature and archwire shape have caused different effects for different brand brackets. From Table 2, two basic principles can be observed. The lager the archwire, the higher FR value; elastic ligature can cause the higher FR values than stainless steel wire except CCB. Higher values could be attributed to the increase of friction factor caused by larger contact areas at the interface of wire-slot and wire-ligature. Meanwhile, it should be noted that different brand of ceramic bracket has a different contour and size. When archwire was ligated in slot, excessive size of bracket and archwire certainly would increase the deformation force of elastic ring and lead to larger vertical pressure for archwire. In comparison, stainless steel wire ligature provides a constant initial force by the same operator.

Slot of CCB was polished with ruby oilstone slice, which tends to cause a sharp corner on slot edges. The excessive force of ligaturing would give rise to some permanent deformations of archwire and form notch, especially for smaller round wires at interface of wire and bracket corner. Tooth movement stops when a notched wire catches on the bracket corner and resumes only when the notch is released [25]. When combined with thinner round wires, the sliding resistance of CCB could attribute to the physical notching and deformation of wire instead of contact friction. Therefore, the FR variation regularity of CCB was inconsistent with that of other three ceramic brackets. Overall, the statistical analyses of FR results with different archwires and ligature combinations suggested that CCB has an approximate frictional property with commercial brackets CA, C7 and IC.

Because ceramic materials do not bond chemically with adhesives, ceramic brackets derive their bond strength from the use of a silane coupling agent on the base of the bracket, through mechanical retention, or both [26]. Therefore, conventional ceramic brackets bases comprise various designs for increasing the surface area. Alumina abrasion blasting was used to divest the investment materials and create an initial morphology of CCB base (Fig. 7a). Although the abrasive blasting process had formed a rough topography on base surface, it still cannot supply cleaned and enough micromechanical bonding (interlocking, retention) surface. After etching, it could be observed that HF acid dissolved glassy contents and exposed the crystal structures (Fig. 7b). Research has shown that silane has the potential to react with hydroxyl groups present on the surface of silica in ceramics and the methacrylate group of bonding agent or resin cement [27]. In this study, microcosmic smoother debonding surface and massive adhesive remnants of CCB-ES (Fig. 7d) supports that stronger chemical bond exist at the interface of ceramic and adhesive, not just some micro-retentions like CCB-E (Fig. 7c).

Some researchers suggested that bond strengths between 6 and 8 MPa are clinically sufficient for bonding of brackets to enamel [28, 29]. And, too high bond strength could result in damage to enamel and existing restorations. Thus, adhesive failures and high ARI score are more favorable to avoid enamel fractures during debonding. In this study, all groups achieved mean values higher than 10 MPa, and no damages were observed to the debonded tooth surface. CCB-E and CCB-ES groups both reached an adequate performance while the bonding and debonding procedures. In addition, CCB base surface allows the treatment of hydrofluoric acid, which gives the ability of clinical re-bonding. Consequently, the CCB system could be considered sufficient and reasonable for clinical applications.

## Conclusion

CCB system makes it possible to realize mass-customization for ceramic orthodontic brackets via personalized design, 3D printing technique, heat-pressing technique, and lithium disilicate materials. Within the limitations of this study, the following conclusions were drawn.

1. CCB attained the multi-color appearance and individual contour by means of lithium disilicate materials and custom molding design.

2. Except 16-SSL group, there were no statistically significant differences between CCB and certain commercial ceramic brackets (*P* ≥ 0.05).

3. The SBS of all groups exhibited higher values than the minimum orthodontic bracket bond strength range of 6–8 MPa, and no statistically significant differences in SBS were observed among CCB-ES, CA and C7 groups (P ≥ 0.05). CCB-E attained the highest mean ARI score 3, and no enamel fractures were detected.

## Data Availability

The data supporting the findings of this research can be obtained directly from the authors as well as the College of materials science and engineering, Sichuan University, Chengdu, China.

## Abbreviations

ARI: Adhesive remnant index
C7: Crystalline VII
CA: Clarity ADVANCED
CCB: customized ceramic bracket
DQ: Damon Q
FR: Frictional resistance
IC: Inspire ICE
SBS: Shear bond strength
